# Phage to ESKAPE: Personalizing Therapy for MDR Infections—A Comprehensive Clinical Review

**DOI:** 10.3390/pathogens14101011

**Published:** 2025-10-07

**Authors:** Andrea Marino, Stefano Stracquadanio, Federica Cosentino, Alberto Enrico Maraolo, Agnese Colpani, Andrea De Vito, Nicholas Geremia, Alice Nicolosi, Alessandra Oliva, Bruno Cacopardo, Giuseppe Nunnari

**Affiliations:** 1 Unit of Infectious Diseases, Department of Clinical and Experimental Medicine, ARNAS Garibaldi Hospital, University of Catania, 95122 Catania, Italy; andrea.marino@unict.it (A.M.); federicacosentino91@gmail.com (F.C.); cacopard@unict.it (B.C.); giuseppe.nunnari1@unict.it (G.N.); 2 Department of Medicine and Surgery, “Kore” University of Enna, 94100 Enna, Italy; 3 Section of Infectious Diseases, Department of Clinical Medicine and Surgery, University of Naples “Federico II”, 80138 Naples, Italy; albertomaraolo@mail.com; 4 Unit of Infectious Diseases, Department of Medicine, Surgery and Pharmacy, University of Sassari, 07100 Sassari, Italy; colpani.agnese@gmail.com (A.C.); andreadevitoaho@gmail.com (A.D.V.); 5 Unit of Infectious Diseases, Department of Clinical Medicine, Ospedale “dell’Angelo”, 30174 Venice, Italy; nicholas.geremia@aulss3.veneto.it; 6 Section of Microbiology, Department of Biomedical and Biotechnological Sciences, University of Catania, 95123 Catania, Italy; alice-nicolosi93@hotmail.it; 7 Department of Public Health and Infectious Diseases, Sapienza University of Rome, 00185 Rome, Italy; alessandra.oliva@uniroma1.it

**Keywords:** bacteriophage therapy, ESKAPE pathogens, antimicrobial resistance (AMR), biofilm, phage–antibiotic synergy

## Abstract

The proliferation of multidrug-resistant (MDR) ESKAPE pathogens—*Enterococcus faecium*, *Staphylococcus aureus*, *Klebsiella pneumoniae*, *Acinetobacter baumannii*, *Pseudomonas aeruginosa*, and *Enterobacter* spp.—constitutes a critical global health crisis, rendering conventional antibiotics increasingly ineffective. This comprehensive review evaluates the re-emerging potential of bacteriophage therapy as a personalized treatment for infections caused by these organisms. Phages, being viruses that specifically infect and lyse bacteria, offer significant advantages, including high specificity that spares host microbiota, self-replication at the infection site, and potent activity against biofilms. This paper synthesizes current preclinical and clinical evidence, including compassionate-use cases, for phage therapy against each of the ESKAPE pathogens. While case reports and small studies demonstrate considerable success, particularly in salvage therapy for otherwise untreatable infections, significant challenges remain. These include the narrow host range of phages, the potential for bacterial resistance, unpredictable pharmacokinetic and pharmacodynamic parameters, and a complex, non-harmonized regulatory landscape. The review highlights that phage–antibiotic synergy and the use of phage cocktails are promising strategies to overcome some of these limitations. Future progress in phage therapy will depend on standardized manufacturing, robust clinical trials to establish dosing and efficacy, and the development of adaptive regulatory pathways. Phage therapy is positioned not as a replacement for antibiotics but as a vital adjunctive tool in the armamentarium against MDR infections, heralding a move towards a more personalized approach to infectious disease management.

## 1. Introduction

The alarming rise of multidrug-resistant (MDR) and extensively drug-resistant (XDR) bacteria represents one of the most pressing public health threats of the 21st century [1]. The global spread of pathogens resistant to multiple classes of antimicrobials has dramatically reduced the efficacy of existing therapies, challenging clinicians and healthcare systems worldwide [2]. According to recent estimates, antimicrobial resistance (AMR) was directly responsible for 1.27 million deaths and associated with nearly 5 million deaths globally in 2019 [1,3], with projections suggesting up to 10 million annual deaths by 2050 if left unchecked [4,5]. Among bacterial pathogens, the so-called ESKAPE group—*Enterococcus faecium*, *Staphylococcus aureus*, *Klebsiella pneumoniae*, *Acinetobacter baumannii*, *Pseudomonas aeruginosa*, and *Enterobacter* spp.—stands out due to their ability to “escape” conventional antibiotic treatment and thrive in nosocomial environments [6,7]. These organisms are now frequent culprits of healthcare-associated infections (HAIs), including bloodstream infections, pneumonia, urinary tract infections (UTIs), and device-related infections, often associated with poor clinical outcomes and increased healthcare costs [8,9].

The discovery and widespread use of antibiotics in the 20th century represented a cornerstone in modern medicine, revolutionizing the management of infectious diseases. However, decades of overuse, misuse (in human health as well as in veterinary and agriculture), and uneven access to antibiotics have fostered the rapid emergence and dissemination of resistance determinants [10]. Simultaneously, the antibiotic development pipeline has dwindled, with few novel compounds reaching the clinic in recent decades [11]. This mismatch between the relentless pace of resistance and the stagnation of therapeutic innovation has renewed interest in alternative and adjunctive approaches to combat bacterial infections. Among these, bacteriophage (phage) therapy—the therapeutic use of viruses that specifically infect bacteria—has re-emerged as a promising strategy [12,13,14].

Phages, first described independently by Frederick Twort in 1915 and Félix d’Hérelle in 1917, are the most abundant biological entities on Earth [15]. They are highly specific viruses that parasitize bacterial hosts, replicating within them and often causing bacterial lysis. Before the advent of antibiotics, phages were used therapeutically in several countries, particularly in Eastern Europe and the former Soviet Union. However, their development in Western medicine was largely abandoned after the widespread availability of antibiotics, which were easier to produce, standardize, and administer. Nevertheless, phage therapy research continued in countries such as Georgia, Poland, and Russia, maintaining decades of clinical experience [16]. With the current AMR crisis, the biomedical community has revisited phages as a potential tool in the antimicrobial armamentarium [17,18].

Phages possess several characteristics that make them attractive as therapeutic agents [19]. First, they exhibit exquisite specificity, often targeting only one species or even particular strains within a species, thus sparing the commensal microbiota. Second, their ability to replicate at the site of infection offers a self-amplifying effect, provided the bacterial host is present. Third, phages can evolve alongside bacteria, potentially counteracting resistance development in a “race for life”. In addition, phage-derived enzymes, such as endolysins and depolymerases, further expand their therapeutic potential [20,21]. These features distinguish phages from traditional antibiotics and open new avenues for personalized and precision-based interventions [19,22].

Nevertheless, phage therapy is not without limitations. The high degree of specificity, while advantageous in terms of microbiome preservation, can limit therapeutic applicability and requires accurate and rapid bacterial identification. The development of bacterial resistance to phages is another concern, though often mitigated by the use of phage cocktails [23,24]. Moreover, regulatory frameworks for phage therapy remain underdeveloped in most parts of the world, complicating standardization, production, and clinical trial implementation [14]. Finally, questions regarding immunogenicity, pharmacokinetics, and interactions with antibiotics remain active fields of investigation [25].

In recent years, an increasing number of compassionate-use cases, clinical trials, and experimental studies have demonstrated both the feasibility and challenges of phage therapy in diverse clinical contexts. Interest from the pharmaceutical industry, regulatory agencies, and research consortia has surged, highlighting the potential of phages to complement or, in selected cases, substitute antibiotics in the treatment of MDR infections [12,13,18,19]. Importantly, several reports describe successful phage use against ESKAPE pathogens, including otherwise untreatable infections, emphasizing the clinical relevance of this approach [24,26].

**Figure 1:**
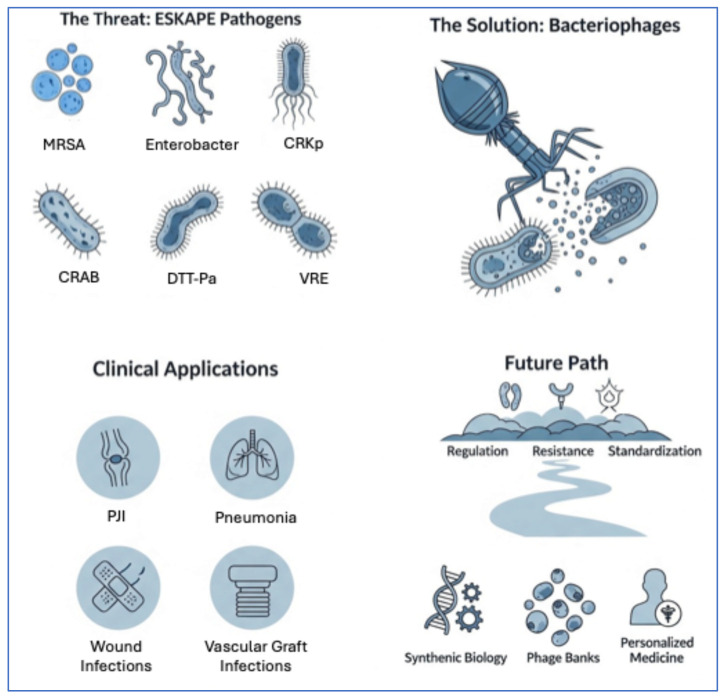
Bacteriophages as a therapeutic strategy against multidrug-resistant ESKAPE pathogens, including methicillin-resistant *Staphylococcus aureus* (MRSA), *Enterobacter* spp., carbapenem-resistant *Klebsiella pneumoniae* (CR-Kp), carbapenem-resistant *Acinetobacter baumannii* (CRAB), difficult-to-treat *Pseudomonas aeruginosa* (DTT-Pa), and vancomycin-resistant *Enterococcus* spp. (VRE). Clinical applications of phage therapy include prosthetic joint infections (PJI), pneumonia, wound infections, and vascular graft infections. Future progress depends on regulation, resistance management, and standardization, supported by synthetic biology, phage banks, and personalized medicine.

This review aims to provide a comprehensive overview of phage therapy, with particular attention to mechanisms of phage action, current evidence from preclinical and clinical studies targeting ESKAPE pathogens, and the challenges that must be addressed to enable their broader implementation (Figure 1). By critically examining both the promises and limitations of phage-based interventions, we aim to contextualize their potential role in future infectious disease management strategies.

## 2. Mechanisms of Phage Action and Therapeutic Potential

Since their discovery, phages have been recognized as the most abundant biological entities on Earth, with an estimated 10^31^ particles globally, vastly outnumbering bacteria and shaping microbial ecosystems. Their ecological role extends beyond bacterial predation, influencing horizontal gene transfer, microbial community composition, and biogeochemical cycling [27].

Historically, phage therapy was pursued in the early 20th century, particularly in Eastern Europe and the former Soviet Union, where phage research centers such as the Eliava Institute in Tbilisi, Georgia, maintained clinical applications despite the rise of antibiotics in the West. The antibiotic boom relegated phages to the margins of Western medicine, but escalating antimicrobial resistance (AMR) has revived global interest in phage-based therapeutics [17].

### 2.1. Phage Isolation and Characterization

Developing therapeutic phages begins with their isolation from environments rich in microbial diversity, such as sewage, soil, or aquatic ecosystems. Classical enrichment techniques, wherein bacterial hosts are exposed to environmental samples to amplify phages, remain foundational. However, modern approaches leverage high-throughput robotics, metagenomics, and culture-independent sequencing to accelerate phage discovery and expand the known phageome [13].

Following isolation, rigorous characterization is critical. Essential parameters include host-range determination, adsorption rate, burst size, and lytic potential. Genomic sequencing ensures the absence of undesirable traits such as lysogeny, toxin genes, or antibiotic resistance determinants. Structural and stability assessments—such as tolerance to pH, temperature, and bile salts—further inform suitability for clinical development [28]. Advanced single-cell and microfluidic assays now allow for rapid profiling of phage-host dynamics at scale, facilitating the rational design of phage cocktails and personalized therapeutic regimens.

### 2.2. Phage Cocktails

A central challenge in phage therapy is the narrow host specificity of individual phages. While this specificity is advantageous in sparing commensal flora, it necessitates combining multiple phages to ensure robust coverage against heterogeneous bacterial populations. Phage cocktails, often composed of 3–10 phages targeting distinct receptors, are designed to mitigate resistance and provide broad-spectrum efficacy [29,30].

Cocktail development involves careful selection of phages with complementary host ranges and non-overlapping resistance profiles. Genomic analysis is crucial to ensure genetic compatibility and to prevent recombination events that could compromise stability. However, the manufacturing of phage cocktails remains complex. Each constituent phage must be cultured, purified (typically via ultrafiltration or chromatography), and characterized individually before blending under stringent Good Manufacturing Practice (GMP) conditions. This complexity increases production costs and creates regulatory hurdles, since cocktail formulations may require adaptation over time as bacterial populations evolve. Nonetheless, advances in modular manufacturing and adaptive licensing models are beginning to address these bottlenecks [31].

### 2.3. Pharmacokinetics, Pharmacodynamics, and Synergy

Phage pharmacokinetics (PK) and pharmacodynamics (PD) remain incompletely characterized in humans. Unlike small-molecule antibiotics, phage efficacy depends on adsorption to susceptible bacteria, with outcomes strongly influenced by multiplicity of infection (MOI) and the local bacterial density. Thus, exposure–response relationships are inherently context-dependent and may vary across infection sites. Local delivery has been crucial in biofilm-rich settings such as prosthetic joint infections (PJIs), while intravenous (IV) administration provides systemic exposure but is subject to rapid clearance by the reticuloendothelial system and potential neutralization by host antibodies [32,33,34].

Nebulized administration achieves high concentrations in the respiratory tract and has shown promising reductions in bacterial load in cystic fibrosis and ventilator-associated pneumonia. Compartmental routes (e.g., intra-articular, intraperitoneal) can maximize exposure at hard-to-reach foci and may overcome biofilm barriers [19,35].

Clearance mechanisms and immunogenicity remain major uncertainties. Circulating phages are eliminated rapidly, and neutralizing antibodies may appear within days of treatment. However, neutralization does not invariably preclude therapeutic success, particularly when high local titers are achieved. Repeated dosing and sequential phage cocktails have been used to sustain activity and overcome emerging resistance. Preclinical studies highlight the importance of antibiotic–phage synergy (PAS), with sequencing effects suggesting that phage-before-antibiotic exposure may optimize biofilm clearance, while antibiotic-first may favor planktonic infections [32,36,37,38].

To translate these insights into clinical practice, future trials should embed PK/PD substudies and prespecified adaptive elements. We recommend serial PK sampling after IV and local dosing (including blood and accessible site fluids), paired with culture-based monitoring to track resistance emergence. Neutralizing antibodies should be assessed at baseline, day 7, day 14, and day 28 to inform dosing intervals and cocktail adaptation. Trial endpoints should be syndrome-specific: ventilator-free days and microbiological clearance for respiratory infections, revision-free survival for prosthetic joint infections, and composite clinical cure for bloodstream infections. Adaptive or platform trial designs allowing sequential cocktail switching will be critical to address resistance and maximize generalizability. In summary, addressing these PK/PD and immunogenicity uncertainties with standardized PK studies, resistance monitoring, and syndrome-specific endpoints will be essential to move from compassionate use toward robust trial evidence.

The gaps identified and the corresponding remedial measures to be undertaken are detailed in Table 1.

### 2.4. Clinical Trials and Animal Models

Clinical evidence for phage therapy, though still limited, is growing. An overview of the currently registered clinical trials is provided in the Supplementary Materials (Table S1). Compassionate-use cases have documented successful treatment of multidrug-resistant infections refractory to antibiotics, including *Klebsiella pneumoniae*, *Pseudomonas aeruginosa*, and *Staphylococcus aureus*. The case of a cystic fibrosis patient with disseminated *Mycobacterium abscessus* treated with engineered phages remains one of the most compelling examples of personalized phage therapy [12].

Preclinical animal studies further validate efficacy across models of sepsis, pneumonia, wound infection, and gastrointestinal colonization. A systematic review and meta-analysis confirmed significant survival benefits in phage-treated animals, with reductions in bacterial burden and inflammatory markers compared to controls [39]. While randomized controlled trials remain scarce, ongoing efforts—such as the PhagoBurn trial in burn wound infections—illustrate both the promise and challenges of transitioning from experimental to standardized clinical practice [18].

### 2.5. Regulatory Landscape

Regulatory approval remains one of the most significant hurdles for phage therapy. In the United States, phages are currently administered primarily under the FDA’s Expanded Access or eIND programs, limiting their use to compassionate cases rather than routine clinical deployment. In Europe, the absence of harmonized guidelines has led to heterogeneity: Belgium’s magistral phage framework permits pharmacies to prepare bespoke formulations, whereas other countries rely on experimental-use exemptions [40].

Globally, the debate centers on whether phages should be regulated as biological medicinal products, advanced therapy medicinal products (ATMPs), or require their own dedicated category. Each option carries implications for manufacturing standards, trial design, and adaptability. Given the evolutionary nature of phages and the need for rapid reformulation, adaptive regulatory frameworks are increasingly recognized as essential. Initiatives such as the European Medicines Agency’s (EMA) concept paper on phage therapy and the establishment of phage biobanks represent significant steps toward standardization [41]. To promote harmonization and facilitate clinical use under compassionate-access frameworks, we suggest the adoption of a minimum regulatory checklist. At a minimum, candidate phages should undergo whole-genome sequencing to exclude lysogeny, toxins, or antimicrobial resistance genes, and stability testing under anticipated storage conditions [42,43]. Identity assays (e.g., spot/host-range testing, PCR) and potency assays (e.g., PFU titers, efficiency of plating against clinical isolates) should be standardized across centers [44,45]. For compassionate-use cases, documentation should include informed consent (explicitly noting investigational status), genomic and phenotypic matching data retained for auditing, and batch-specific sterility and endotoxin results [13]. Harmonization of these basic requirements would allow cross-country comparability, support regulators in evaluating expanded access or magistral-use applications, and give clinicians clearer frameworks for safe, transparent practice. Table 2 presents the steps that need to be taken into account for the implementation of phage therapy.

Beyond regulatory harmonization, cost and scalability remain major barriers. Production of individualized phage cocktails requires complex upstream (isolation, sequencing, characterization) and downstream (purification, QC) workflows, with associated expense and time constraints. Single-phage preparations are cheaper to manufacture and quality-control, but cocktails are often necessary to address bacterial heterogeneity and prevent resistance. Centralized manufacturing facilities or regional ‘phage banks’ with pre-characterized libraries may reduce costs and enable rapid access, but investment in scalable production pipelines is essential for widespread clinical uptake [46].

### 2.6. Future Perspectives

The trajectory of phage therapy is poised to benefit from a confluence of technological innovation, translational initiatives, and regulatory reform. Synthetic biology and genetic engineering are at the forefront of efforts to reprogram receptor-binding proteins and expand host specificity, deliver antimicrobial payloads, and harness artificial intelligence (AI)-based design approaches, which are already underway [47,48].

Notably, CRISPR-enhanced bacteriophages represent a cutting-edge innovation: companies like Locus Biosciences have advanced CRISPR-Cas3 phage cocktails into Phase 2 clinical trials, offering promise against MDR infections such as *E. coli* UTIs, and early trials utilizing CRISPR-augmented phages show encouraging results in reducing *E. coli* levels.

AI further augments this progress by enabling more efficient, data-driven phage–host pairing and therapeutic design. Recent perspectives underscore how machine learning can guide synthetic phage development, while new AI models increasingly predict effective phage combinations from genomic data [49].

Translational efforts and regulatory adaptations are evolving in parallel. In the EU, emerging legal frameworks are debating the categorization of phage products, and recent European Commission initiatives, including an EMA concept paper and pharmacopoeia chapters, indicate growing institutional readiness for phage-based medicinal products [41,50]. Similarly, compassionate-use programs such as UC San Diego’s IPATH and FDA expanded-access eINDs continue to provide critical patient access while generating valuable safety data.

Bacteriophage therapy is re-emerging as a credible countermeasure to MDR bacterial infections. While technological advances in isolation, characterization, and synthetic design are accelerating progress, challenges remain in manufacturing, regulation, and large-scale clinical validation. Integrating phages into mainstream medicine will require interdisciplinary collaboration, harmonized policies, and robust clinical trials. With continued investment and innovation, phage therapy is poised to evolve from an experimental intervention into a cornerstone of 21st-century antimicrobial strategies.

## 3. Phage Therapy Against Individual ESKAPE Pathogens (Preclinical and Clinical Trials/Experiences)

### 3.1. Enterococcus faecium

*Enterococcus faecium* is a Gram-positive, facultatively anaerobic coccus belonging to the Enterococcaceae family, widely recognized as a commensal of the human gastrointestinal tract but also as an opportunistic pathogen in healthcare settings [51,52]. Among the enterococci, *E. faecium* has emerged as a leading cause of nosocomial infections, particularly in immunocompromised patients. The threat posed by this pathogen lies in its ability to acquire MDR determinants [53,54]. For this reason, the global spread of vancomycin-resistant *E. faecium* (VREfm) has been classified by the WHO as a high-priority public health threat, due to its limited therapeutic options and high mortality rates in bloodstream, urinary tract, and device-associated infections [55,56,57].

Conventional management of *E. faecium* infections relies on antibiotics such as linezolid, daptomycin, and vancomycin. However, increasing reports of reduced susceptibility or outright resistance to these agents highlight the need for alternative or adjunctive therapies [55,57]. Among these, bacteriophage therapy is drawing growing attention as a potential strategy against MDR and VREfm infections.

Phage therapy research has shown promising results in vitro, ex vivo, and in vivo, with phages demonstrating potent bactericidal activity, synergy with antibiotics, and biofilm-disrupting properties. For example, the recently characterized *E. faecium* phage EF-M80 exhibits a broad host range, stability across varying environmental conditions, and strong biofilm degradation that make it a strong therapeutic candidate [58]. Similarly, phage cocktails combining multiple phages have proven effective in controlling *E. faecium* growth, including strains resistant to individual phages or antibiotics. This approach broadens host coverage and can slow the development of resistance [59,60,61,62,63].

The catalogue of *E. faecium*-targeting phages includes siphovirus-type phages (e.g., EfV12-phi1) and myoviruses (e.g., MDA2, Porthos, iF6), many of which retain activity against VREfm [59,62,64]. These diverse morphologies and receptor specificities are valuable for designing cocktails tailored to clinical isolates.

Real-world applications of *E. faecium* phage therapy are rare but offer encouraging insights. In one pediatric case, intravenous administration of a personalized phage (EFgrKN) alongside vancomycin not only reduced intestinal colonization but also shifted the infecting strain toward vancomycin susceptibility [65]. This resulted in fewer hospitalizations; unfortunately, the patient died from pneumonia unrelated to the initial infection. In another example, compassionate phage therapy for an infected vascular graft was well tolerated in an adult female, with no adverse events reported [66]. In this case, the patient had recurrent VREfm bacteremia who, after failing multiple antibiotics, received an intravenous phage-antibiotic combination, with a rapid bacteremia clearance and several months of infection-free survival before recurrence, likely due to a combination of bacterial adaptation and anti-phage antibody development.

Mechanistically, phage adsorption in *E. faecium* depends on surface structures such as wall teichoic acids, lipoteichoic acid, and capsular polysaccharides [67,68]. Variability in these components explains the narrow host range of many phages and highlights the importance of maintaining diverse phage libraries for clinical use. Resistance can arise through receptor modification or immune neutralization, but such adaptations may incur fitness costs, reducing virulence or restoring antibiotic susceptibility [68,69].

Biofilm-associated infections, common in *E. faecium*, pose a particular challenge. Phages equipped with depolymerases can degrade biofilm matrices, improving both phage penetration and antibiotic access. This dual action offers a strategic advantage in managing chronic or device-associated infections.

Despite promising data, challenges remain. Host-range limitations necessitate rapid diagnostic tools to match patients with effective phages. Immune responses, including neutralizing antibodies, can shorten treatment effectiveness. Regulatory and manufacturing hurdles continue to slow clinical adoption. Finally, robust clinical trials are needed to validate safety, efficacy, and optimal use protocols for *E. faecium* phage therapy. Recent studies concerning the use of phages for the treatment of *E. faecium* infections are reported in Table 3.

### 3.2. Staphylococcus aureus

*Staphylococcus aureus* is one of the most formidable human pathogens, responsible for severe disease ranging from bacteremia, endocarditis, prosthetic joint infections (PJIs), to fracture-related infections (FRIs). The rise of methicillin-resistant *S. aureus* (MRSA) and the biofilm-forming ability of this organism complicate management, often rendering conventional antibiotic therapy inadequate. In this context, phage therapy has re-emerged as a potential adjunctive or salvage option [70].

The available literature over the last few years illustrates the growing role of phage therapy as a powerful adjunctive and salvage modality. A significant portion of this evidence centers on complex orthopedic device-related infections, where biofilms render conventional treatments inadequate [71].

Recent studies have demonstrated the potential of intra-articular phage therapy in conjunction with debridement and implant retention (DAIR). Ferry et al. introduced the “PhagoDAIR” approach, wherein three elderly patients with recurrent prosthetic knee infections were treated with intra-articular phage cocktails in combination with antibiotics. All patients exhibited clinical improvement, regained mobility, and remained free from relapse, although minor persistent synovial leakage was observed in two cases [72]. The synergy of surgical debridement and sustained phage pressure may be crucial in some instances: the case detailed by Ramirez-Sanchez et al. provides a compelling narrative of this approach, where an initial two-week course of a phage cocktail failed, but a subsequent, more aggressive strategy involving a two-stage surgical revision and a prolonged six-week course of a single lytic phage (administered both via intravenous and intra-articular route) led to a durable cure of a persistent methicillin-sensitive *S. aureus* (MSSA) PJI [73]. The potential of even short-course therapy was highlighted by Doub et al. (2020), who reported the successful clearance of a chronic MRSA PJI after just three days of intravenous phage therapy, which stopped for an unexpected but reversible increase in transaminase levels, suggesting that rapid bacterial load reduction is achievable [74]. Preclinical validation in a rabbit model of FRI further supports these findings, demonstrating that a single intraoperative dose of phages in saline was highly effective in preventing infection [75]. However, treating an established FRI in the same model proved more complex, with a single application of a phage-loaded hydrogel showing only a modest, non-significant trend toward improvement over antibiotics alone [75].

The therapeutic application of bacteriophages is experiencing significant growth beyond the field of orthopedics. A case series conducted by Rubalskii et al. (2020) demonstrated the effectiveness of personalized phage preparations in eight patients suffering from critical infections associated with cardiothoracic surgery. The study achieved bacterial eradication in seven cases and introduced the innovative use of fibrin glue as a local phage delivery system [76]. The successful salvage of a complex *S. aureus* extradural empyema with local phages and dalbavancin further demonstrates its utility in sensitive and hard-to-reach sites [77].

The potential application of phage therapy in the treatment of chronic rhinosinusitis was evidenced in a patient with a multi-year refractory MRSA infection. Although initial intravenous and topical therapies yielded partial benefits, a significant and sustained clinical response, accompanied by negative cultures, was only achieved following the implementation of a regimen involving frequent, daily topical administration. This outcome highlights the necessity of attaining high local phage concentrations for the effective management of localized infections [78].

In a particularly severe case, a 12-year-old boy with life-threatening necrotizing fasciitis caused by Panton–Valentine Leukocidin (PVL)-producing MRSA that was failing maximal standard of care, was rescued with a personalized, multi-route (intravenous, topical, intra-pleural, intra-peritoneal, nebulized) phage therapy targeting not only *S. aureus* but also subsequent superinfecting pathogens, leading to a full recovery [79].

Looking forward, Doub et al. identified spinal epidural abscesses (SEAs) as a prime candidate for future trials, as the near-universal association of *S. aureus* SEAs with bacteremia offers a practical method for isolating the target pathogen from blood cultures to guide phage selection [80].

Despite this growing list of successes, the literature also highlights critical challenges.

A primary concern is the host immune response. The preclinical FRI study conducted by Onsea et al. demonstrated that repeated local administration of phages elicited neutralizing antibodies, whereas encapsulation within a hydrogel appeared to protect the phages from the immune system [75]. However, clinical success in the PJI case reported by Ramirez-Sanchez et al. was achieved despite the development of serum neutralization, suggesting its clinical impact may be variable [73]. In the above-mentioned pediatric case of necrotizing fasciitis also noted the development of a phage immune neutralization (PIN) response that did not preclude a positive outcome [79].

The complexity of polymicrobial infections was highlighted by Van Nieuwenhuyse et al., where a “kill-the-leader” strategy targeting only *S. aureus* in a bone allograft biofilm led to short-term improvement but ultimate relapse, emphasizing the need for comprehensive therapeutic coverage [81].

The interaction between bacteriophages and co-administered antibiotics presents a particularly complex challenge. The in vitro study conducted by Loganathan et al. offers significant insights, indicating that the optimal sequence of administration may vary depending on the type of infection. Specifically, for planktonic (acute) bacterial infections, administering antibiotics prior to phages proved most effective. Conversely, in the case of biofilms (chronic infections), administering phages before antibiotics resulted in the highest survival rates, as demonstrated in a *Galleria mellonella* model [82].

Finally, the question of safety, especially with systemic administration, is paramount. This concern was directly addressed in a landmark single-arm trial by Petrovic Fabijan et al., where 13 critically ill patients with severe *S. aureus* bacteremia and endocarditis received intravenous phage therapy. The study’s primary outcome was met, concluding that the GMP-quality phage cocktail was safe and well-tolerated, with no adverse reactions attributed to the infusions [83]. Table 4 presents recent studies on the use of phages in the treatment of *S. aureus* infections.

### 3.3. Klebsiella pneumoniae

*Klebsiella* spp. is a Gram-negative, facultatively anaerobic commensal bacterium belonging to the *Enterobacterales* family [84,85]. Among these, *Klebsiella pneumoniae* is the most challenging species due to its remarkable ability to produce a protective capsule, making it particularly difficult to eradicate through host immune responses and antibiotic therapy [86]. Antibiotic resistance is one of the most challenging topics in *K. pneumoniae*, where the expression of different resistant mechanisms can confer a wide range of resistance to the most common antibiotics [87,88]. Moreover, in recent years, persistent and resistant infections caused by hypervirulent *K. pneumoniae* have emphasized its growing significance in global public health, concerning an important issue related to fatal cases and the risk of biofilm-forming infections [87,88,89].

Although *K. pneumoniae* represents a major problematic human pathogen, conventional management relies on increasingly advanced antibiotics. Introducing novel molecules has undoubtedly improved clinical outcomes [90]. Although this antibiotic-based approach remains the cornerstone of therapy and should be pursued as first-line treatment, microbiological eradication is not always achievable, particularly in patients with chronic or relapsing infections. In such scenarios, phage therapy has emerged as a promising adjunctive strategy [86].

Currently, phage therapy is mainly employed in critical or compassionate-use settings, often in cases where conventional antibiotic regimens have failed [86,88]. In recent years, many case reports and small clinical series have documented the efficacy of phage therapy against *K. pneumoniae* infections, underscoring the increasing interest in this therapeutic approach [86,91]. Documented successes include treating recurrent UTIs with phage cocktails, pneumonia, inflammatory bowel disease, and PJIs complicated by biofilm formation [92,93,94].

An illustrative example of the efficacy and anti-biofilm activity of phage therapy involved a 62-year-old man with chronic prosthetic knee infection caused by MDR *K. pneumoniae*, refractory to multiple antibiotic regimens and surgeries. Intravenous administration of a single phage (KpJH46Φ2) over 40 doses, in combination with minocycline, resulted in clinical resolution, functional recovery, and no recurrence at 34-week follow-up, with in vitro confirmation of anti-biofilm activity [92]. Moreover, a retrospective study of 12 patients receiving personalized phage therapy for resistant infections, including *K. pneumoniae*, reported favorable outcomes in 66% without serious adverse events. Notably, this review showed a case of a transplant recipient with recurrent urosepsis due to *K. pneumoniae*. After the treatment, the *K. pneumoniae* persisted in the urine, but the bacteremia resolved with no further episodes of sepsis in over 1 year since phage therapy [19]. A systematic review further noted that phage therapy, usually combined with antibiotics, achieved clinical efficacy in 87% of published ESKAPE infection cases, including *K. pneumoniae*, with an acceptable safety profile. Overall, in many of these cases, a partial or complete clinical resolution was observed, supporting the therapeutic potential of phages [91].

In many of these cases, a partial or complete clinical resolution was observed, supporting the therapeutic potential of phages [91].

In vivo studies using mouse models have also produced encouraging results. However, most preclinical investigations have focused on acute systemic infections like pneumonia and bacteremia. This creates a translational gap, as in clinical practice, phage therapy has thus far been more commonly applied to chronic infections, including urinary tract and joint infections [86,91,95,96,97].

A key virulence factor of *K. pneumoniae* is its capsular polysaccharide (CPS), which plays a central role in immune evasion and antimicrobial resistance [98]. The CPS comprises repeating oligosaccharide units that constitute the K-antigen, which defines the K-type of the strain [91,98]. Another vital surface structure is the lipopolysaccharide (LPS), consisting of lipid A, a core oligosaccharide, and a variable O-antigen, which is used to classify strains by O-type [99].

An essential step in developing phage-based treatments is the selection of suitable phages, which is determined mainly by their host range. Most phages targeting *K. pneumoniae* are specific for particular K-types, a feature that represents a double-edged sword: on the one hand, this narrow specificity allows for precise bacterial targeting and reduces the risk of cross-resistance; on the other hand, the vast diversity of K-types significantly limits the breadth of coverage of any single CPS-targeting phage. To address this limitation, growing attention is being directed toward CPS-independent phages, which recognize more conserved bacterial structures—such as O-antigens or surface-associated proteins—thus enabling a broader host range and increasing therapeutic applicability across diverse strains [91].

Resistance to phage predation is often cited as a significant obstacle to the success of phage therapy, especially because it can emerge more rapidly than resistance to conventional antibiotics, particularly in vitro. Nevertheless, in some cases, the development of phage resistance has been linked to reduced virulence or altered resistance profiles, potentially resulting in significant clinical benefit even in the absence of complete bacterial eradication [91,100]. Moreover, phage resistance can alter bacterial susceptibility patterns, occasionally increasing sensitivity to other phages within a therapeutic cocktail [86,91,101].

Therefore, even when phage therapy fails to eliminate *K. pneumoniae*, it may still reshape the bacterial population, steering it toward a less virulent and more treatable phenotype. However, while this evolutionary trade-off can be advantageous, unintended consequences may also arise. Resistance mechanisms that involve modification or loss of CPS may enhance horizontal gene transfer, increasing the risk of acquiring additional antibiotic resistance genes via conjugation [102]. Additionally, acapsular variants may develop enhanced tolerance to membrane-targeting antimicrobial peptides, enabling bacterial persistence or regrowth even under high-dose antibiotic therapy [86,103]. Recent investigations addressing the application of phages for the treatment of *K. pneumoniae* infections are summarized in Table 5.

### 3.4. Acinetobacter baumannii

*Acinetobacter baumannii* remains a formidable pathogen in hospital environments, particularly due to its extensive drug resistance and ability to persist on abiotic surfaces. It frequently causes ventilator-associated pneumonia, bloodstream infections, and wound infections, especially among immunocompromised or critically ill patients [104]. The World Health Organization has classified carbapenem-resistant *A. baumannii* as a critical priority pathogen for the development of new antimicrobials [105]. Its resistance to last-line antibiotics such as colistin, alongside β-lactams and fluoroquinolones, has severely limited therapeutic options [104].

Over the past decade, significant progress has been made in the experimental use of phages against *A. baumannii*, with results that support their therapeutic potential.

Preclinical models have consistently demonstrated the efficacy of phage therapy in improving survival and reducing bacterial burden. A recent Bayesian meta-analysis encompassing various animal models reported survival rates in phage-treated groups between 60% and 90%, compared to 20–50% in untreated controls. In murine models, survival following phage treatment exceeded 96% on average [106]. These results suggest that phages can substantially improve outcomes in severe infections caused by MDR *A. baumannii*.

Therapeutic benefits extend beyond increased survival. In one study using the myovirus vB_AbaM_Acibel004, mice infected with *A. baumannii* exhibited reduced lung pathology, lower systemic and local inflammatory cytokines, and preservation of lung architecture following phage administration [107]. Nebulized phage delivery has also proven effective in respiratory infection models. A single-dose nebulized formulation containing the phage vB_AbaM_PhT2 achieved complete bacterial clearance and 100% survival in mice when administered immediately after infection [108]. The treatment also led to significant reductions in TNF-α, IL-6, and IFN-γ levels, indicating potent anti-inflammatory effects in the lung.

Adjunctive use of phage-derived enzymes has shown additional promise. A purified depolymerase (Dpo48), capable of degrading the capsular polysaccharide of *A. baumannii*, significantly improved survival in *Galleria mellonella* larvae when administered either before or after infection. In murine models, Dpo48 demonstrated no detectable toxicity, further supporting its potential as a phage adjunct to enhance bacterial clearance [109].

While these results are encouraging, the translation of phage therapy into clinical practice remains in early stages. However, several case reports and small-scale studies have provided compelling evidence of efficacy in humans. A notable example involved a 68-year-old patient with a life-threatening *A. baumannii* infection following necrotizing pancreatitis [13]. The isolate was resistant to all tested antibiotics, and the infection persisted despite multiple interventions. A custom phage cocktail was administered intravenously and later intraperitoneally. Although resistance emerged to the initial phage formulation, a second cocktail was successfully applied, leading to resolution of the infection.

Similar success has been observed in treating respiratory infections. In one case, a patient with chronic obstructive pulmonary disease and ventilator-associated pneumonia caused by carbapenem-resistant *A. baumannii* was treated with nebulized phages [110]. After 16 days of therapy, bacterial clearance was achieved and respiratory function improved, without any reported adverse effects. In a separate report, four patients with COVID-19 developed secondary pneumonia due to *A. baumannii* while on mechanical ventilation [111]. Phage therapy was administered via vibrating-mesh nebulizer, resulting in bacterial clearance in three patients and clinical improvement in two. Phage resistance was observed during treatment in some cases, underscoring the need for adaptive or sequential phage strategies.

Taken together, recent developments in phage therapy against *A. baumannii* highlight its potential as a viable alternative or adjunct to antibiotic treatment. The current body of evidence, though still limited in scale, demonstrates that phage therapy can achieve clinical success where antibiotics fail. Continued research is now focused on refining delivery systems, expanding phage libraries, and conducting controlled clinical trials to determine optimal treatment regimens [111]. Given the urgency of antimicrobial resistance and the lack of new antibiotics in the pipeline, phage therapy represents a promising and timely avenue for addressing the growing burden of MDR *A. baumannii* infections. Table 6 outlines recent studies regarding the therapeutic use of phages against *A. baumannii* infections.

### 3.5. Pseudomonas aeruginosa

*Pseudomonas aeruginosa* is an opportunistic Gram-negative pathogen responsible for severe infections in immunocompromised individuals, particularly in hospital-acquired pneumonia, cystic fibrosis (CF), and wound and bloodstream infections [112]. Its remarkable ability to form biofilms and acquire multidrug resistance (MDR) makes treatment highly challenging [113]. Phage therapy has therefore re-emerged as a promising alternative or adjunctive strategy in such scenarios [114].

Evidence of efficacy largely derives from infections where biofilms compromise standard therapies, including orthopedic device-related infections, vascular graft infections, and CF pneumonia [115,116,117].

Recent reviews have emphasized the therapeutic potential of phages targeting *P. aeruginosa*, supported by in vitro, in vivo, and compassionate-use case studies [113,114,118,119].

PAS further enhances efficacy, with studies showing improved bacterial clearance, biofilm disruption, and reduced resistance when phages are combined with sub-inhibitory antibiotic doses [120,121]. PAS has been particularly relevant in CF lung infections [122].

A targeted literature review of 23 studies on MDR *Pseudomonas aeruginosa*-related lung infections indicates that phages can effectively lyse antibiotic-resistant strains and disrupt biofilms in vitro. In vivo and clinical use showed improvements in disease markers and survival rates, with no significant adverse events reported. Synergistic interactions were observed between phages, host immunity, and antibiotics, supporting their therapeutic potential [114].

Khosravi et al. highlighted the advantages of phage therapy in overcoming biofilm-mediated resistance, while also noting major challenges that must be addressed before it can be integrated into mainstream respiratory care, including regulatory hurdles, production standardization, and trial design [123].

Clinical evidence, though limited, is encouraging. A placebo-controlled trial in the UK demonstrated the safety and efficacy of a phage cocktail in chronic *P. aeruginosa* otitis [124]. Over the 42-day follow-up, phage-treated patients showed significant improvements in both clinician- and patient-reported symptoms, as well as marked reductions in bacterial counts. In some cases, persistent bacterial clearance was achieved. No adverse events were reported. Other trials reported mixed outcomes, with efficacy often limited by low phage titers or bacterial resistance [18]. More recently, nebulized phages (BX004-A) in CF patients showed significant bacterial clearance. The study demonstrated the safety and tolerability of the phage cocktail, supporting progression to Phase 2b trials [125].

Currently, phage therapy is mainly employed in critical or compassionate-use settings, typically in cases where conventional antibiotic regimens have failed.

Large multicenter series reported clinical improvement in over 70% of patients and bacterial eradication in ~60%, especially when combined with antibiotics [126]. Programs such as PHAGEinLYON in France have shown the feasibility of structured phage deployment in hospital settings [127].

Case reports in orthopedic, vascular, and prosthetic infections highlighted durable infection clearance when phages were used adjunctively with antibiotics.

Cesta et al. reported a case in Italy of a chronic hip prosthetic infection caused by *P. aeruginosa*, treated with personalized phage therapy (phage Pa53 administered intra-articularly via joint drainage) combined with meropenem, following DAIR. The intervention was well tolerated, and after two years, sustained infection clearance was achieved with no relapse. In vitro, the phage and meropenem exhibited synergy in eradicating established biofilms, a combination that was ineffective when used individually at the same concentrations. This case highlights the potential of adjunctive phage-antibiotic therapy in managing recalcitrant biofilm-associated infections [128].

The potential use of phage therapy for chronic vascular graft infections was illustrated in a patient with chronic *Pseudomonas aeruginosa* endocarditis following a Bentall procedure, where surgical revision carried excessive risk. Phage therapy led to infection resolution and relapse-free survival at 12 months, demonstrating the potential of phages in managing high-risk prosthetic device infections when conventional options fail [129].

Despite promising results, significant challenges remain. Limitations include phage resistance, serum neutralization, declining titers, and incomplete efficacy against biofilm-associated infections. Reports of treatment failures, particularly in ventricular assist device infections, underline the need for optimized dosing strategies, improved stability, and better integration with antibiotics [130].

In summary, phage therapy—especially in synergy with antibiotics—holds substantial potential for managing MDR *P. aeruginosa* infections. However, its translation into routine clinical practice requires rigorous clinical trials, regulatory standardization, and further mechanistic insights into biofilm eradication and host–phage interactions. Recent research exploring the therapeutic use of phages against *P. aeruginosa* infections is presented in Table 7.

### 3.6. Enterobacter *spp.*

Phage therapy is emerging as a rational adjunct or alternative to antibiotics for difficult *Enterobacter* infections—particularly those caused by carbapenem-resistant members of the *Enterobacter cloacae* complex (ECC)—because lytic phages can be selected to match a patient’s isolate, kill rapidly without collateral dysbiosis, and retain activity in biofilms that blunt antibiotic efficacy. Recent preclinical and translational data specific to *Enterobacter* are encouraging. In 2025, Fu and colleagues reported an ECC-targeted cocktail for carbapenem-resistant *E. cloacae* bacteremia, showing how dosing and early administration shape outcomes; their work underscores that timely, sufficiently high multiplicities of infection can widen the therapeutic window and limit resistance, offering practical guidance for compassionate-use protocols [131].

In parallel, multiple groups have expanded the phage toolbox against ECC: new strictly lytic *E. cloacae* phages lacking lysogeny, toxin, or AMR genes (e.g., ECLFM1) have been genomically vetted and characterized, while additional phages targeting *E. kobei* and *E. hormaechei* broaden strain coverage across the complex [28,132,133].

These inputs matter clinically because ECC causes healthcare-associated urinary, bloodstream, and device-related infections with high rates of carbapenemase production and biofilm formation [134].

Biofilm-focused experiments that included *Enterobacter* isolates on catheter materials suggest that properly matched lytic phages can markedly reduce established biofilm biomass—an effect likely to translate to urologic devices where ECC thrives [135].

Beyond single-agent activity, PAS is increasingly documented against *E. cloacae*, where specific antibiotic classes potentiate phage replication and phage exposure resensitizes bacteria to drugs via fitness trade-offs in surface receptors; this supports combination designs that limit resistance and improve sterilization of deep-seated foci [120].

At the bedside, species-specific clinical evidence for *Enterobacter* remains limited compared with *Pseudomonas* or *Staphylococcus*, but the broader ECC-relevant UTI space is moving into prospective evaluation. A multi-site U.S. Phase 1/2 study of an expanding PhageBank™ for UTIs is now enrolling, a format suited to *Enterobacterales* etiologies and individualized matching; along with a registered trial of phages for UTIs, these efforts will yield safety/efficacy data and inform regulatory pathways applicable to *Enterobacter* [136].

Case series from expanded access programs also describe clinical responses to personalized, multi-route phage therapy across Gram-negative infections, reinforcing feasibility and the value of combination approaches, although species-resolved outcomes for *Enterobacter* are still sparse [137].

Methodologically, modern phage practice emphasizes genomic vetting (to exclude lysogeny/AMR cargo), pharmacology (high local titers; repeated dosing to overcome clearance and neutralizing antibodies), and in vitro matching assays with the patient isolate; recent state-of-the-art reviews codify these principles and highlight manufacturing and regulatory advances that lower barriers to responsible use [19].

For *Enterobacter* specifically, two challenges recur. The first involves host-range gaps: ECC serovar/CLT diversity and rapid receptor evolution demand larger, curated libraries and rational cocktails; institutional efforts are now designing frontline ECC cocktails from genomically diverse phages to pre-empt resistance [138].

The second challenge is durability in UTIs: while phages reach high titers in urine and can be instilled locally, variable success in early clinical attempts likely reflects lysogeny in endogenous urinary phages, immune neutralization, and complex bladder ecology—issues motivating engineered or sequential regimens and rigorous susceptibility testing [139].

Overall, the 2024–2025 literature supports a cautious but optimistic stance: high-quality ECC phages exist; biofilm and PAS data are favorable; dosing and timing rules of thumb are crystallizing; and prospective UTI trials with library-based matching are underway. For clinicians considering phage therapy against *Enterobacter* today, best practice is to (i) obtain the patient isolate(s) for rapid phage susceptibility testing against a well-annotated library; (ii) co-design an antibiotic backbone expected to produce PAS (e.g., β-lactams or agents that perturb outer-membrane targets implicated in phage adsorption); (iii) favor high local exposure (e.g., bladder instillation or intra-cavity delivery when applicable) plus systemic dosing for invasive disease; and (iv) monitor for anti-phage antibodies and emergent resistance with serial cultures, adjusting the cocktail as needed. As regulatory-grade manufacturing and trial infrastructure mature, *Enterobacter*-targeted phage therapy is poised to move from bespoke salvage to protocolized adjunctive care, especially for carbapenem-resistant ECC infections associated with devices or persistent bacteremia [140,141]. An outline of recent studies addressing phage-based treatment of *Enterobacter* infections is given in Table 8.

## 4. Challenges and Future Perspectives

Although phage therapy represents a promising strategy to treat antimicrobial-resistant infections caused by ESKAPE pathogens, several uncertainties and challenges must be addressed.

First of all, only lytic phages can be considered for phage therapy. Secondly, phages are species-specific and even strain-specific. Although this is considered an advantage in terms of preserving human microbiota, it in turn means that phages present a very narrow spectrum of action [142].

Evolution plays a key role in the phage–bacteria interaction. Bacteria can develop mutations and strategies that render them resistant to phage infection, with such traits being naturally selected due to their evolutionary advantage; phages can evolve alongside. Although phage resistance has been demonstrated in vitro, clinical studies have shown both the presence and absence of this phenomenon [143]. When resistance emerges, it often comes with a fitness cost or attenuated virulence [13]. The challenge of host resistance to the phages can be overcome by using multiple phages (i.e., phage cocktail) with the aim of targeting multiple receptors simultaneously and broadening the antibacterial spectrum. Phages can also be genetically engineered using various approaches [144].

Another concern is the patient’s immune response, especially the production of neutralizing antibodies. It is not yet clear whether these antibodies could compromise the therapeutic outcome. For instance, the presence of antibodies was associated in some cases with a loss of clinical response [145], while in others it did not result in therapeutic failure [146]. Looking ahead, further research is needed to investigate the impact of the patient’s immune response on phage efficacy and to determine a standardized dosage that ensures therapeutic effectiveness without triggering antibody production.

Currently, phage therapy can be administered in two ways: as an industrial product or through a magistral, personalized approach [31]. In the industrial approach, individual phages or phage cocktails are commercialized as medicinal products and are therefore subject to extensive, time-consuming, and costly requirements prior to market authorization, including GMP certification, preclinical and clinical trials, and favorable assessments from regulatory agencies. In contrast, the personalized or tailored approach involves selecting phages specifically targeting the bacterial strain(s) responsible for the infection in a given patient. This approach requires precise characterization of the pathogen’s strain before administering the specific phage. This distinction in therapy administration helps explain the discrepant outcomes observed between randomized controlled trials (RCTs), which often reported disappointing results, and observational studies or case reports employing the personalized approach, which generally showed more favorable outcomes. Several factors may account for these differences: (i) the time frame from phage development to market entry can allow the bacterial host to evolve and develop resistance; (ii) challenges exist in developing phages or phage cocktails targeting multiple bacterial species and strains, including those that will be clinically relevant at the time of market release; and (iii) due to the high costs involved, commercially available phages or cocktails typically target only a limited number of species for which broadly active phages are available. In contrast, the personalized approach employs phages specifically active against the well-characterized infecting strain in the individual patient.

All this considered, one of the main obstacles to the clinical implementation of phage therapy is the regulatory framework [147]. Indeed, high heterogeneity among different countries can be observed. As an example, in the United States, phage therapy is classified as a biological product and regulated accordingly, while in Italy (our country of reference), there is no specific legislation regarding phage therapy, and its use is limited to compassionate cases as a personalized strategy. In contrast, the regulatory approach in Belgium and Georgia is significantly more favorable. In Belgium, phage therapy is regulated as a magistral preparation, meaning that pharmacists can produce custom phage cocktails under medical prescription. In Georgia, where phage therapy has been a part of the healthcare practice since its discovery, the pre-prepared phage medicines are under legislation for market authorization, while personalized phage medicines are allowed to be manufactured in pharmacies specially licensed by the Georgian Ministry of Healthcare through magistral preparation. To solve this heterogeneity at the European level, a general chapter has recently been published in the European Pharmacopoeia to establish the framework for phage therapy [148].

Despite these challenges, phage therapy offers unique opportunities, making it a promising alternative or complement to traditional antibiotics, particularly in the context of increasing antimicrobial resistance. At present, the largest real-life experience with the use of personalized phage therapy for the treatment of infections caused by difficult-to-treat microorganisms includes 100 patients from multiple centers across Europe [126]. In most cases, phage therapy was administered as a salvage treatment following the failure of standard antibiotic therapy. In approximately half of the patients, the pathogens were MDR, and in 69% of cases, phages were used in combination with antibiotics. Overall, clinical improvement and microbiological eradication were achieved in approximately 77% and 61% of cases, respectively. High tolerability of phage therapy was also demonstrated: the adverse events described as severe (*n* = 7) were all considered likely unrelated to the administration of phage therapy, which was, nevertheless, discontinued in all subjects [126].

This is an important aspect, since one of the main concerns in the field of phage therapy is related to safety, especially after intravenous administration, which is employed less frequently due to the potential risk of hypersensitivity reactions possibly resulting from the rapid release of bacterial endotoxins or cellular components following bacterial lysis [142]. Although high safety of phage therapy can be expected since phages are directed exclusively against the bacterial host and not against human eukaryotic cells, further research on a large number of patients is needed to confirm this aspect.

Another relevant factor favoring the implementation of phage therapy research is the relatively low production cost: phages can be isolated from sources such as soil, wastewater, and feces. Their production is relatively simple and cost-effective, and can even be carried out in resource-limited settings [149].

Considering the global rise in antimicrobial resistance, the development of complementary therapeutic strategies to conventional antibiotic therapy has become an urgent priority. Within this framework, phage therapy, especially when administered as a personalized approach, represents one of the most promising and encouraging advances, as demonstrated by the growing number of clinical experiences in difficult-to-treat infections, including those associated with biofilms or caused by MDR bacteria [126,128,150]. Exploring bacteriophages as a promising non-antibiotic strategy to eradicate carriage of multidrug-resistant bacteria without globally disrupting the microbiota may also be considered, although clinical experience remains largely anecdotal [151].

It is therefore essential to harmonize and regulate access to such therapy not only at the European level but also within individual countries. Looking ahead, the establishment of phage banks or repositories appears crucial for the advancement of phage therapy, as they could enable the administration of well-characterized bacteriophages—both at molecular and phenotypic levels—specifically targeting the infecting strain.

Nevertheless, the relatively small number of patients treated to date underscores the existing knowledge gap regarding the optimal way of administration and appropriate dosing of phages based on pharmacokinetic/pharmacodynamic (PK/PD) considerations. This highlights the need for further research and international consensus efforts aimed at standardizing phage therapy across countries.

Ethical considerations warrant particular attention, too. As a matter of fact, in the context of compassionate use and adaptive phage switching, issues such as informed consent, equitable access to phage therapy, and transparent data sharing should be systematically addressed. Ensuring robust ethical frameworks will indeed be crucial to align the clinical implementation of phage therapy with patients’ rights and regulatory standards.

Table 9 outlines the benefits and limitations of phage therapy.

## 5. Conclusions

Phage therapy is poised to evolve from bespoke salvage into protocolized adjunct care for difficult-to-treat infections caused by ESKAPE pathogens, supported by growing preclinical efficacy, compassionate-use successes, biofilm activity, and frequent phage–antibiotic synergy alongside an encouraging safety signal when products are well characterized and matched to the patient’s isolate. Yet, broad implementation still hinges on three priorities: (i) standardizing selection, dosing, and PK/PD-informed delivery; (ii) expanding curated, genomically vetted phage libraries and accessible phage banks to enable timely personalization; and (iii) harmonizing regulatory frameworks to accommodate adaptive and magistral preparations while ensuring quality, traceability, and comparability across centers. In the near future, best practice couples individualized phages with active antibiotics, integrates rapid susceptibility testing and monitoring for resistance or neutralizing antibodies, and favors high local exposure for biofilm-dense foci. In this regard, key research priorities in phage therapy include (i) standardization of clinical protocols; (ii) randomized clinical trials targeting specific conditions such as device-associated infections; (iii) comprehensive evaluation of phages’ safety and efficacy; and (iv) development of standardized large-scale production methods for the establishment of phage banks and (iv) standardization of PK/PD methods.

Looking ahead, synthetic biology (including depolymerases and CRISPR-enhanced phages) and AI-guided matching promise broader host range and greater durability but must be validated in rigorous multicenter trials embedded within antimicrobial stewardship programs. Given the accelerating burden of AMR and the early clinical gains already documented, investment in phage infrastructure, clinical trial networks, and pragmatic policy is a timely, pragmatic path to safer, more effective management of MDR infections—placing phages alongside, not in place of, antibiotics.

## 6. Materials and Methods

This review was conceived as a narrative, comprehensive overview of the current evidence regarding bacteriophage therapy against ESKAPE pathogens. We did not perform a systematic review, as our goal was to integrate data from diverse sources (including case reports, preclinical studies, compassionate-use experiences, and clinical trials) in order to provide a clinically oriented synthesis. We searched PubMed/MEDLINE, Embase, and ClinicalTrials.gov databases for relevant publications from January 2000 through January 2025. The following search terms, alone or in combination, were used: bacteriophage therapy, phage therapy, multidrug-resistant bacteria, antimicrobial resistance, ESKAPE pathogens, *Enterococcus faecium*, *Staphylococcus aureus*, *Klebsiella pneumoniae*, *Acinetobacter baumannii*, *Pseudomonas aeruginosa*, *Enterobacter* spp. Only articles in English were included. Reference lists of relevant reviews and case series were also screened for additional studies. The last search was run on 31 January 2025. We included preclinical models (in vitro and in vivo), clinical trials, case reports, and case series describing phage therapy targeting ESKAPE pathogens. Exclusion criteria were studies not focused on bacteriophage therapy, non-ESKAPE pathogens, or articles lacking original data (e.g., editorials, commentaries). For clarity, case reports and case series were categorized as compassionate-use experiences; randomized or prospective interventional studies were labeled as clinical trials; and non-randomized human studies (e.g., single-arm safety studies) were classified separately. Preclinical studies were divided into in vitro experiments and animal models. This classification was maintained in the tables and narrative synthesis to ensure transparency. Across ESKAPE pathogens, we found 3 randomized controlled trials (all *P. aeruginosa*), 1 prospective single-arm safety study (*S. aureus*), 4 case series, and 13 case reports, plus 2 prospective non-randomized studies and 1 retrospective observational series in *Enterobacterales*/*Enterobacter*-focused cohorts. Evidence is therefore heavily weighted toward uncontrolled, compassionate-use reports, especially for *E. faecium*, *K. pneumoniae*, and *A. baumannii*, which increases the risk of publication bias and limits efficacy inference; by contrast, only *P. aeruginosa* currently has randomized data.

To provide a comparative overview, we summarized the available clinical evidence for each pathogen in a consolidated master table (Table 10).

## Figures and Tables

**Table 1:** Practical translational gaps and recommended experiments for clinical phage therapy.

Gap	Why It Matters	Recommended Experiment/Plan
Human PK by route (IV, nebulized, local)	Dosing remains empirical; clearance and tissue penetration are poorly defined	Embed PK substudies in early-phase trials: serial blood and site sampling (PFU + qPCR) to model clearance and local persistence
MOI/exposure targets at infection site	Required titers for effective kill are undefined, especially in biofilm	Dose-escalation with local sampling (e.g., BAL, synovial fluid) to link input dose → site PFU → bacterial kill
Biofilm penetration and PAS sequencing	Order of phage–antibiotic administration may alter outcomes	Factorial preclinical/early clinical studies comparing phage→antibiotic vs. antibiotic→phage sequences with quantitative microbiology
Immunogenicity and neutralization kinetics	Neutralizing antibodies may reduce efficacy	Prospective neutralization assays (baseline, day 7, day 14, day 28) with prespecified cocktail switch criteria
Resistance emergence and management	On-therapy resistance can derail efficacy	Adaptive protocols enabling sequential cocktail switching upon phenotypic resistance; genomic analysis of resistant isolates
Syndrome-specific endpoints	Heterogeneous outcomes obscure efficacy signals	Core outcome sets: ventilator-free days + culture negativity for VAP; revision-free survival for PJI; symptom + culture clearance for otitis/burns

**Table 2:** Practical regulatory checklist for hospital-based phage use programs.

Domain	Minimum Recommendation
Phage characterization	Whole-genome sequencing; exclude lysogeny, toxins, AMR genes
Quality control	Sterility testing (bacteria, fungi, mycoplasma); endotoxin quantification; stability testing at intended storage conditions
Identity assays	Host-range determination (efficiency of plating), sequence-based ID, qPCR
Potency assays	PFU titers per batch; in vitro lytic activity against patient isolate
Documentation for compassionate use	Informed consent (investigational status, risks, uncertainty); clinical isolate susceptibility records; batch QC data retained for inspection
Regulatory filings	eIND or Expanded Access (US), magistral preparation (Belgium), or local equivalents
Manufacturing standards	GMP-like or magistral-level oversight; validated sterility and stability protocols

**Table 3:** Key information from recent studies on phage therapy for *E. faecium* infections.

Study (First Author, Year)	Infection Type	Study Design	Key Pathogen(s)	Phage Administration Details	Key Outcomes & Findings
Paul et al., 2021 [65]	VREfm infection post–liver transplant (pediatric)	Case report	*E. faecium* (VRE)	Personalized phage EFgrKN IV + vancomycin	Reduced intestinal colonization; shift toward vancomycin susceptibility; fewer hospitalizations; death from unrelated pneumonia
Stellfox et al., 2024 [66]	Recurrent VREfm bacteremia	Case report	*E. faecium* (VRE)	IV phage + antibiotics	Rapid bacteremia clearance; infection-free for months before recurrence likely due to adaptation and anti-phage antibodies
Khazani Asforooshani et al., 2024 [58]	Wound infection model	Preclinical (in vivo)	*E. faecium*	Hydrogel-encapsulated phage EF-M80 (topical)	Broad host range; biofilm degradation; accelerated wound healing features
Pradal et al., 2023 [62]	Sepsis model (Galleria)	Preclinical (in vivo)	*E. faecium* (vanR clinical isolate)	vB_EfaH_163	Reduced mortality vs. control
Wandro et al., 2019 [59]	Coevolution/host-range	Preclinical (in vitro)	*E. faecium*	Lytic phage EfV12-phi1	Predictable molecular adaptation; informs cocktail design
Lossouarn et al., 2024 [63]	Host-range expansion	Preclinical (in vitro)	*E. faecium* (VRE)	Appelmans training method	Extended host spectra against VREfm strains
Ghatbale et al., 2025 [64]	PAS/resensitization	Preclinical (in vitro)	*E. faecium/E. faecalis*	Phage + antibiotic combinations	Resensitized MDR isolates; supports PAS in treatment design

**Table 4:** Key Information from recent studies on phage therapy for *S. aureus* infections.

Study (First Author, Year)	Infection Type	Study Design	Key Pathogen(s)	Phage Administration Details	Key Outcomes & Findings
Ferry et al. (2020) [72]	Prosthetic Knee Infection (PKI)	Case Series (*n* = 3)	*S. aureus*	Local intra-articular injection of phage cocktail post-DAIR surgery (“PhagoDAIR”).	Favorable outcomes with significant clinical improvement; potential as a salvage therapy.
Bleibtreu et al. (2020) [77]	Extradural Empyema	Case Report	*S. aureus* (MSSA)	Local administration of two phages via fistula, combined with IV dalbavancin.	Successful salvage therapy, stopping purulent flow and allowing for reconstructive surgery; culture-negative samples post-treatment.
Ramirez-Sanchez et al. (2021) [73]	Prosthetic Knee Infection (PJI)	Case Report	*S. aureus* (MSSA)	Two cycles: 1st (cocktail, 2 weeks) failed; 2nd (single phage, 6 weeks, intravenous and intra-articular) with surgery succeeded.	Highlights the importance of surgical debridement and sufficient duration. Success was achieved despite the development of serum neutralization.
Rubalskii et al. (2020) [76]	Cardiothoracic Surgery-Related Infections	Case Series (*n* = 8)	*S. aureus,* *P. aeruginosa, E. faecium,* *K. pneumoniae, E. coli*	Individualized phage preparations via local, oral, or inhalation routes. Three cases used a fibrin glue delivery system.	Eradication of target bacteria in 7 of 8 patients as a last resort therapy.
Onsea et al. (2021) [75]	Fracture-Related Infection (FRI)	Preclinical (Rabbit)	*S. aureus*	Prevention: Single intraoperative phage in saline. Treatment: Daily phage in saline vs. single phage-loaded hydrogel.	Highly effective in prevention. In treatment, hydrogel showed a trend but no statistical significance. The hydrogel avoided the antibody response seen with saline application.
Van Nieuwenhuyse et al. (2021) [81]	Polymicrobial Bone Allograft Infection	Case Report	*S. aureus,* *C. hathewayi,* *P. mirabilis,* *F. magna*	Local, in situ anti-	*S. aureus* phage therapy for 14 days.
Doub et al. (2023) [80]	Spinal Epidural Abscess (SEA)	Feasibility Study	*S. aureus*	N/A (in vitro testing only).	More than 50% of the patients either succumbed within three months, experienced a recurrence of their infection, required additional debridement, or suffered long-term sequelae.
Doub et al. (2020) [74]	Prosthetic Joint Infection (PJI)	Case Report	*S. aureus* (MRSA)	Intra-articular and 3 days of IV phage therapy.	Successful eradication of chronic infection despite a short course, which was stopped due to reversible transaminitis.
Petrovic Fabijan et al. (2020) [83]	Severe Bacteremia and Endocarditis	Single-Arm Safety Trial (*n* = 13)	*S. aureus* (MSSA & MRSA)	Intravenous administration of Good Manufacturing Practices-quality phage cocktail AB-SA01 twice daily for 14 days.	Primary outcome met: Therapy was safe and well-tolerated in critically ill patients, with no reported adverse reactions.
Van Nieuwenhuyse et al. (2024) [79]	Necrotizing Fasciitis (Pediatric)	Case Report	PVL-producing MRSA, *P. aeruginosa, S. maltophilia*	Personalized, multi-route (intravenous, topical, intra-pleural, etc.) therapy targeting all three pathogens, combined with antibiotics.	Successful rescue of a life-threatening infection failing standard care. Phage immune neutralization was observed.
Rodriguez et al. (2022) [78]	Chronic Rhinosinusitis (CRS)	Case Report	*S. aureus* (MRSA)	Two courses of IV and topical phage. Dramatic improvement seen only with frequent, daily topical (intranasal/ear) application.	Successful treatment of a multi-year refractory infection, highlighting the importance of achieving high local phage concentrations.
Loganathan et al. (2024) [82]	MRSA Biofilm Infection	In vitro & In vivo (Larvae)	*S. aureus* (MRSA)	Tested three different treatment sequences: phages before, with, or after antibiotics.	Confirmed synergy with multiple antibiotics. Phages before antibiotics (PRE) were most effective against biofilms; antibiotics before phages (POS) were most effective against planktonic cells.

**Table 5:** Key Information from recent studies on phage therapy for *K. pneumoniae* infections.

Study (First Author, Year)	Infection Type	Study Design	Key Pathogen(s)	Phage Administration Details	Key Outcomes & Findings
Cano et al., 2021 [92]	Prosthetic knee infection (biofilm)	Case report + in vitro	*K. pneumoniae*	Personalized phage therapy adjunctive to DAIR; local delivery	Limb-threatening PJI salvaged; anti-biofilm activity shown in vitro
Bao et al., 2020 [93]	Recurrent UTI (XDR)	Case report	*K. pneumoniae* (XDR)	Phage + “non-active” antibiotic	Successful eradication via synergism
Le et al., 2023 [94]	Recurrent drug-resistant UTI	Prospective series	*Enterobacterales* incl. *K. pneumoniae*	Intravenous phage as a standalone	Demonstrated safety/therapeutic potential for recurrent UTIs
Tang et al., 2024 [95]	Hypervirulent infection (hvKP)	Preclinical	Hv *K. pneumoniae*	Phage therapy with immune analyses	Host immunity engagement influenced outcomes
Rahimi et al., 2023 [5]	Pneumonia (murine model)	Preclinical (in vivo)	K2 hv *K. pneumoniae*	Phage PSKP16	Therapeutic efficacy in mice
Kelishomi et al., 2024 [97]	Infected wounds (murine)	Preclinical (in vivo)	*K. pneumoniae*	Novel phage topical/application	Improved wound healing parameters
Li et al., 2023 [100]	MDR infection under personalized therapy	Case report	*K. pneumoniae* (MDR)	Personalized phage	Phage resistance emerged with reduced virulence; clinical improvement despite resistance

**Table 6:** Key information from recent studies on phage therapy for *A. baumannii* infections.

Study (First Author, Year)	Infection Type	Study Design	Key Pathogen(s)	Phage Administration Details	Key Outcomes & Findings
Malik et al., 2025 [106]	Various animal infections	Meta-analysis (preclinical)	*A. baumannii* (MDR)	Multiple	Survival 60–90% with phage vs. 20–50% controls; >96% mean survival in murine models
Wienhold et al., 2021 [107]	Experimental lung infection	Preclinical (murine)	*A. baumannii*	Myovirus vB_AbaM_Acibel004	Reduced lung pathology/inflammation; preserved architecture
Sitthisak et al., 2023 [108]	Respiratory infection model	Preclinical	*A. baumannii*	vB_AbaM_PhT2 endolysin ± colistin	Complete bacterial clearance/100% survival in immediate dosing; strong anti-inflammatory effects (model)
Liu et al., 2019 [109]	Systemic infection models	Preclinical (Galleria & mice)	*A. baumannii*	Depolymerase Dpo48	Improved survival; no detectable toxicity in mice
Schooley et al., 2017 [13]	Disseminated, pan-resistant infection	Case report	*A. baumannii*	Personalized cocktail IV → intraperitoneal; sequential adaptation	Resolution after the second cocktail; showcases an adaptive phage strategy
Tan et al., 2021 [110]	COPD patient, CRAB VAP	Case report	*A. baumannii* (Carbapenem resistant)	Nebulized phages	Bacterial clearance after 16 days; respiratory improvement; no adverse effects
Tan et al., 2023 (review) [111]	COVID-19 secondary VAP (*n* = 4)	Case series (summarized)	*A. baumannii*	Nebulized via a mesh nebulizer	Clearance in 3/4; clinical improvement in 2; resistance observed in some

**Table 7:** Key information from recent studies on phage therapy for *P. aeruginosa* infections.

Study (First Author, Year)	Infection Type	Study Design	Key Pathogen(s)	Phage Administration Details	Key Outcomes & Findings
Wright et al., 2009 [124]	Chronic otitis	Randomized, placebo-controlled	*P. aeruginosa*	Topical phage cocktail	Symptom and bacterial-count improvement over 42 days; no AEs
Jault et al., 2019 [18]	Burn wounds (PhagoBurn)	Randomized, double-blind Phase 1/2	*P. aeruginosa*	Topical phage cocktail	Mixed efficacy; issues with low titers highlighted
Cesta et al., 2023 [128]	Hip prosthetic joint infection	Case report + in vitro	*P. aeruginosa*	Intra-articular phage Pa53 via drain + meropenem	Two-year relapse-free clearance; phage–meropenem synergy vs. biofilm
Eiferman et al., 2025 [129]	Chronic endocarditis (post-Bentall)	Case report	*P. aeruginosa*	Personalized phage therapy	Infection resolution; 12-month relapse-free survival
Weiner et al., 2025 [125]	CF chronic lung infection	Randomized first-in-human trial	*P. aeruginosa*	Nebulized cocktail BX004-A	Significant bacterial clearance; safety/tolerability; progressed development
Aslam et al., 2024 [130]	VAD-associated infections (*n* = 5)	Case series (negative)	*P. aeruginosa*	Various	Ineffective outcomes; underscores the need for dosing/PK optimization

**Table 8:** Key information from recent studies on phage therapy for *Enterobacter* infections.

Study (First Author, Year)	Infection Type	Study Design	Key Pathogen(s)	Phage Administration Details	Key Outcomes & Findings
Fu et al., 2025 [131]	Carbapenem-resistant *E. cloacae* bacteremia	Clinical study (dose/timing optimization)	*E. cloacae* complex	Targeted cocktail; early, high MOI dosing	Dosing & early administration shape outcomes; practical guidance for compassionate use
Ali et al., 2024 [132]	CR-*E. cloacae*	Preclinical (genomics + in vitro)	*E. cloacae*	vB_EclM_ECLFM1 (strictly lytic)	Genomically vetted; therapeutic potential against CR strains
González-Gómez et al., 2024 [134]	—	Preclinical (genomic/biological)	*E. cloacae*	Multiple novel phages	Characterized phages vs. high-priority pathogen
Pintor-Cora et al., 2025 [133]	Food/environment relevance	Preclinical	*E. kobei* (mcr-9)	Novel phage	Potential application; broadens ECC coverage
Cieślik et al., 2025 [135]	Urological catheter biofilm	Preclinical (device model)	*E. hormaechei*	Lytic phages on catheter	Marked reduction/elimination of established biofilm
Adaptive Phage Therapeutics, 2023 [136]	Urinary tract infections	Phase 1/2 (enrolling)	*Enterobacterales* incl. *Enterobacter*	Expanding PhageBank™; personalized matching	Will generate safety/efficacy data applicable to *Enterobacter* UTIs
Green et al., 2023 [137]	Various Gram-negatives	Retrospective expanded-access series (*n* = 12)	Mixed (incl. *Enterobacterales*)	Customized phages; multi-route	Feasibility & clinical responses; species-resolved data for *Enterobacter* are still limited

**Table 9:** Pros and cons of phage-based therapy.

Advantages	Challenges
Highly present in nature and easy to isolate (i.e., from soil, wastewater)	Only lytic bacteriophages can be used in therapy
Self-replicating and self-limiting	Bacteriophages need to be isolated and checked for activity before therapeutic use
Low production costs	Narrow spectrum of action
Highly specific against the bacterial host	Possibility of phage resistance in the host
Rapid bacterial killing	Development of neutralizing antibodies
Activity against MDR pathogens	Unpredictable PK/PD parameters
Anti-biofilm activity	Uncertainty in dosages and duration of therapy
Synergism with antibiotics	Safety issues (especially if IV)
No activity against human microbiota	Regulatory heterogeneity among countries
No activity against eukaryotic cells	
Multiple ways of administration (local, aerosol, oral, intravenous)	

**Table 10:** Summary of clinical evidence for bacteriophage therapy against ESKAPE pathogens. * Levels of evidence based on Oxford Centre for Evidence-Based Medicine: Level 1 = RCT; Level 2 = controlled cohort/single-arm prospective; Level 3 = retrospective cohort; Level 4 = case series/case report.

Pathogen	Study (First Author, Year)	Design	N (Patients)	Route(s) of Administration	Primary Endpoint(s)	Key Outcome	Level of Evidence *
*E. faecium*	Paul, 2021 [65]	Case report (compassionate use)	1	IV + antibiotics	Safety, clearance	Clinical improvement, shift to vancomycin susceptibility	Level 4
*E. faecium*	Stellfox, 2024 [66]	Case report	1	IV + antibiotics	Safety, clearance	Rapid bacteremia clearance, relapse-free interval	Level 4
*S. aureus*	Petrovic Fabijan, 2020 [83]	Single-arm safety study	13	IV phage cocktail	Safety	Well tolerated, no adverse reactions	Level 2b
*S. aureus*	Ferry, 2020 [72]	Case series	3	Intra-articular	Clinical resolution	All improved, no relapse at follow-up	Level 4
*K. pneumoniae*	Cano, 2021 [92]	Case report	1	IV phage + DAIR	Infection clearance	Sustained resolution at 34 weeks	Level 4
*K. pneumoniae*	Bao, 2020 [93]	Case report	1	IV phage + ‘inactive’ antibiotic	Clearance	Successful eradication	Level 4
*A. baumannii*	Schooley, 2017 [13]	Case report (adaptive cocktail)	1	IV → intraperitoneal	Survival	Resolution after second cocktail	Level 4
*A. baumannii*	Tan, 2021 [110]	Case report	1	Nebulized	Clearance	Bacterial clearance, clinical improvement	Level 4
*P. aeruginosa*	Wright, 2009 [124]	RCT (placebo-controlled)	24	Topical	Safety, bacterial counts	Improved symptoms and clearance	Level 1b
*P. aeruginosa*	Jault, 2019 [18]	RCT (Phase 1/2)	27	Topical	Safety, efficacy	Safe but efficacy limited (low titers)	Level 1b
*P. aeruginosa*	Cesta, 2023 [128]	Case report	1	Intra-articular + meropenem	Clearance	2-year relapse-free cure	Level 4
*Enterobacter* spp.	Fu, 2025 [131]	Clinical study (dose/timing optimization)	Not specified	IV cocktail	Survival, clearance	Favorable with early/high dosing	Level 3b

## Data Availability

No new data were created.

## Supplementary Materials

The following supporting information can be downloaded at: https://www.mdpi.com/article/10.3390/pathogens14101011/s1, Table S1: Registered ongoing clinical trials involving phage therapy.
