# Supplementary material for: Phage to ESKAPE: Personalizing Therapy for MDR Infections—A Comprehensive Clinical Review
Source: Pathogens. 2025 Oct 7;14(10):1011. doi: 10.3390/pathogens14101011 (PMC12566643; doi:10.3390/pathogens14101011)
Supplement: Supplementary file 1 [file pathogens-14-01011-s001.zip › pathogens-3900110-supplementary.pdf]

ClinicalTrial.gov ID	Title	Enrollment status	Type of infection
NCT04650607	Phage Safety Cohort Study	Recruiting	Serious infections
NCT06368388	Bacteriophage Therapy for Difficult-to-treat Infections: the Implementation of a Multidisciplinary Phage Task Force (PHAGEFORCE)	Recruiting	Difficult-to-treat musculoskeletal infections, chronic rhinosinusitis, sepsis, pulmonary infections associated with cystic fibrosis or bronchiectasis, or hidradenitis suppurativa
NCT06409819	Phage Therapy for Recurrent UTIs in Kidney Transplant Recipients	Recruiting	Asymptomatic female Kidney Transplant Recipients with a history of Recurrent UTIs
NCT05948592	Bacteriophage Therapy TP-102 in Patients With Diabetic Foot Infection (REVERSE2)	Recruiting	Diabetic foot infection
NCT06870409	Bacteriophages in Addition to Antibiotics for the Treatment of Patients With Infective Endocarditis (PHARAOH)	Recruiting	Infective Endocarditis
NCT06998043	Study With Phage for CF Subjects With *Pseudomonas* Lung Infection	Recruiting	CF Subjects With *Pseudomonas* Lung Infection
NCT07076238	Biomarker Investigation of Response to Bacteriophage Treatment for Bacterial Infection	Recruiting	Nontuberculous Mycobacterial Lung Disease
NCT06185920	PHAGEinLYON Clinic Cohort Study: a Descriptive Study of Severe Infections Treated With Phage Therapy at the HCL.	Recruiting	Severe infections
NCT05488340	A Study of LBP-EC01 in the Treatment of Acute Uncomplicated UTI Caused by Drug Resistant E. Coli (ELIMINATE Trial) (ELIMINATE)	Recruiting	Acute Uncomplicated UTI Caused by Drug Resistant *E. Coli*
NCT06319235	Clinical Trial to Demonstrate the Safety and Efficacy of DUOFAG®	Recruiting	Surgical site infections caused by *S. aureus and P. aeruginosa.*
NCT06262282	Mycobacteriophage Treatment of Non-tuberculosis Mycobacteria (POSTSTAMP)	Enrolling by invitation	Non-tuberculosis Mycobacteria
NCT03140085	Bacteriophages for Treating Urinary Tract Infections in Patients Undergoing Transurethral Resection of the Prostate	Completed	Urinary tract infection (UTI)
NCT04803708	Bacteriophage Therapy TP-102 in Diabetic Foot Ulcers (REVERSE)	Completed	Infected and non- infected diabetic foot ulcers with *P. aeruginosa, S. aureus* and/or *A. baumanni*
NCT05616221	Study to Evaluate the Safety, Phage Kinetics, and Efficacy of Inhaled AP-PA02 in Subjects With Non-Cystic Fibrosis Bronchiectasis and Chronic Pulmonary *Pseudomonas Aeruginosa* Infection (Tailwind)	Completed	Non-Cystic Fibrosis Bronchiectasis and Chronic Pulmonary *P. aeruginosa* Infection
NCT05453578	A Phase 1b/2 Trial of the Safety and Microbiological Activity of Bacteriophage Therapy in Cystic Fibrosis Subjects Colonized With *Pseudomonas aeruginosa*	Completed	Cystic Fibrosis Subjects Colonized With *Pseudomonas aeruginosa*
NCT05010577	Nebulized Bacteriophage Therapy in Cystic Fibrosis Patients With Chronic *Pseudomonas aeruginosa* Pulmonary Infection	Completed	Cystic Fibrosis Patients With Chronic *Pseudomonas aeruginosa* Pulmonary Infection
NCT04596319	Ph 1/2 Study Evaluating Safety and Tolerability of Inhaled AP-PA02 in Subjects With Chronic Pseudomonas Aeruginosa Lung Infections and Cystic Fibrosis (SWARM-Pa)	Completed	Chronic *Pseudomonas aeruginosa* Lung Infections and Cystic Fibrosis
NCT05184764	Study Evaluating Safety, Tolerability, and Efficacy of Intravenous AP-SA02 in Subjects With *S. aureus* Bacteremia (diSArm)	Completed	*S. aureus* Bacteremia
NCT05177107	Bacteriophage Therapy in Patients With Diabetic Foot Osteomyelitis (DANCE)	Terminated	Diabetic foot osteomyelitis
NCT04682964	Bacteriophage Therapy in Tonsillitis	Active, not recruiting	Acute tonsillitis
NCT07048704	Taking Advantage of Phage Technologies (TAPT) to Facilitate Phage Therapy While Reducing the Use of	Not yet recruiting	Cystic Fibrosis (CF)

	Antibiotics in the Management of Cystic Fibrosis (CF)		
NCT07202234	Bacteriophage Cocktail Therapy for Multidrug-Resistant Gram-Negative Ventilator-Associated Pneumonia	Not yet recruiting	Multidrug-Resistant Gram-Negative Ventilator-Associated Pneumonia
NCT06605651	Proof of Concept Study to Assess Safety and Efficacy of Phage Therapy in Hip or Knee Prosthetic Joint Infections Due to Staphylococcus Aureus Treated by DAIR. (GLORIA)	Not yet recruiting	Hip or Knee Prosthetic Joint Infections
NCT06370598	Phase 1/2a to Assess the Safety and Tolerability of TP-122A for the Treatment of Ventilator-Associated Pneumonia (RECOVER)	Not yet recruiting	Ventilator-Associated Pneumonia

Table S1. Registered ongoing clinical trials involving phage therapy
